# PCB-defect: An annotated dataset for surface defect detection in printed circuit boards

**DOI:** 10.1016/j.dib.2025.112296

**Published:** 2025-11-26

**Authors:** Ahmed Jawad Rashid, Mohammad Aman Ullah, Adiba Isfara, Nadim Ahmed, Md. Mamun Mian, Md. Mashur Shalehin

**Affiliations:** Department of Electrical and Electronic Engineering, Islamic University of Technology, Gazipur 1704, Bangladesh

**Keywords:** Printed circuit boards, Computer vision, Fault diagnosis, Deep learning

## Abstract

The PCB-Defect dataset was developed to advance automated defect detection in Printed Circuit Boards. This dataset presents a comprehensive collection of 230 annotated high-resolution images of single-layer PCBs, manufactured through a controlled laboratory process. The curated diversity in PCB layouts, defect types, image quality, and annotation density supports the development of robust computer vision models for defect detection. Each board was fabricated using chemical etching on FR4 substrates to introduce manufacturing defects, including missing pad, mouse bite, open circuit, short circuit, spur, and spurious copper. The defect types were embedded at the design phase, physically realized during etching, and confirmed post-manufacturing. Images range from 800 × 600 to 6000 × 4000 pixels, averaging 6.61 megapixels. Detailed bounding box annotations for all visible defect instances were produced using the Roboflow tool, resulting in a total of 1704 annotated defects across the dataset with about 7.4 annotation per image. Annotations follow the COCO (Common Objects in Context) JSON format, which includes detailed metadata and precise localization of each defect by category and bounding box coordinates. This dataset offers significant reuse potential for both academic and industrial research communities focusing on automated optical inspection, quality control, and transfer learning applications*.*

Specifications Table

**Table:** 

Subject	Computer Sciences
Specific subject area	*Fault Diagnosis of Printed Circuit Boards*
Type of data	.jpg (raw images of defect added single layer pcb), ∗.json (annotations for 6 types of defect).
Data collection	PCB images were obtained by scanning the single layer PCB using the HP ScanJet Pro 3600 f1 Flatbed scanner after boards were fabricated by chemical etching on FR4 substrates. Six defect types were introduced at design stage using ECAD software, realized physically, then verified post-manufacturing. Images were manually annotated for defects using Roboflow • Resolution (DPI): 1600 • Color mode: 24-bit color (True Color) • Exposure/Brightness: Default automatic scanner setting • Scanning area: 5 × 8 inches • File format: JPEG • Image correction: No de-skew or auto enhancements
Data source location	Islamic University of Technology, Gazipur 1704, Bangladesh.
Data accessibility	Repository name: Mendeley Data Data identification number: 10.17632/vdj74sngvn.1 Direct URL to data: https://data.mendeley.com/datasets/vdj74sngvn/1
Related research article	None

## 1 Value of the Data

• The data has 230 annotated images, which have about 7.4 annotations each, and has fine-grained bounding box annotations of six important PCB defect categories: missing pad, open circuit, short circuit, spur, spurious copper, and mouse bite. This comprehensive annotation approach enables researchers to develop and evaluate multi-class defect detection models, addressing the limitation of existing datasets that may only annotate each PCB with a single fault type even though in real manufacturing conditions, multiple faults may exist at the same time.
• The data set contains images of high-resolution variation of 2.57 to 31.26 megapixels (average 6.61 megapixels) and the dimensions vary between 800 × 600 to 6000 × 4000 pixels.
• Compared to existing datasets, such as DeepPCB, which consists of numerous smaller cropped images derived from very high-resolution sources, our dataset offers the advantage of whole-board coverage, preserving global PCB layout context alongside local defect features.
• While the dataset primarily focuses on single-layer PCBs fabricated under controlled laboratory conditions, which may differ from multi-layer or large-scale industrial manufacturing processes, it nevertheless offers detailed real defect annotations and high-resolution images.

## 2 Background

Printed circuit board (PCB) is made up of various discrete layers of conductive copper middled with insulation substrate, forming the framework that supports and interconnects electronic components [1]. Precise circuit patterns are then formed using photolithography and chemical etching followed by the application of a solder mask and silkscreen to protect sensitive areas and facilitate assembly [2]. To ensure each PCB layer meets design specifications, inspection systems must detect various faults such as under- or over-etching causing open or short circuits, multilayer misalignment leading to unintended shorts, and solder mask defects like smudges or scratches that expose or cover pads, resulting in missing connections or unwanted copper [2]. As consumer devices continue to shrink and their designs grow ever more intricate, the ability to detect even the smallest abnormalities, including micro-cracks in traces or sub-pixel mask defects is essential to ensuring performance, reliability and safety in large-scale production lines [3].

The dataset was motivated by the recognition that existing PCB defect datasets often use simplified or synthetic fault representations, such as digital edits or small cropped samples, that fail to capture the full complexity of real PCB production [4,5]. To address these gap, our dataset was created using a controlled chemical etching process that mirrors photolithography and etching [2]. Prior datasets include paired template and test images with annotations for six defect types but rely on pixel-level alignment and binarization, which oversimplifies lighting and ignores full-board context [4]. Compared to existing PCB datasets such as DeepPCB, which contains 1500 pairs of high-resolution images originally sized around 16,000 × 16,000 pixels and clipped into smaller aligned sub-images of 640 × 640 pixels, our dataset offers complementary strengths [4]. While DeepPCB consists of many smaller sub-images focused on defect patches, our dataset includes 230 high-resolution whole PCB images ranging approximately from 1500 × 1200 pixels up to nearly 6000 × 5000 pixels, with multiple annotated defects per image (on average 7.4), reflecting multi-defect distributions. This whole-board coverage combined with six defect types produced through physical chemical etching distinguishes our dataset by allowing models to learn both local defect features and global PCB layout context. Many also limit resolution, use grayscale or binary images, and annotate only single defects per board, though actual faults often co-occur and span multiple layers. Thus, a need exists for a comprehensive, high-resolution dataset featuring authentic fabrication, multi-defect annotations, and natural morphological variations to aid development of robust defect detection models.

## 3 Data Description

**Fig. 1:**
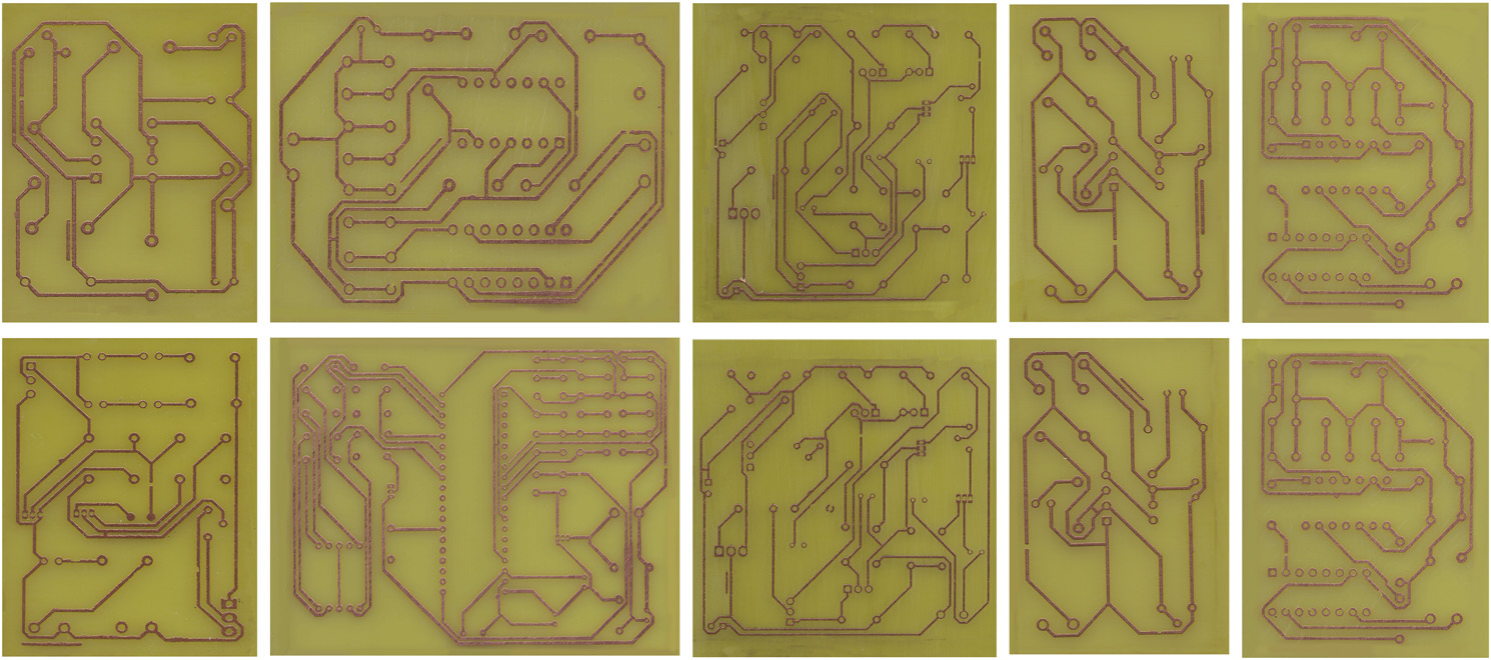
Sample images from PCB defect detection dataset.

The PCB Defect Detection dataset is a collection of real-world PCB images with authentic, chemically induced defects. A total of 230 images were manufactured, each representing a unique PCB manufactured using a controlled chemical etching process. This method ensures that the defects closely mimic those found in industrial settings, including missing pad, mouse bite, open circuit, short, spur, and spurious copper. Each image is annotated with bounding boxes for all visible defect instances, resulting in an average of approximately 7.4 annotations per image with a total of 1704 annotations across 6 defect classes. The dataset covers a wide range of PCB layouts, component densities, and defect scenarios, making it highly representative of actual manufacturing conditions (see Fig. 1 for sample images from the PCB-Defect Dataset). The PCB Defect Detection dataset is publicly available in Mendeley Data [6].

The images in the dataset exhibit a broad range of native resolutions, reflecting the variability encountered in practical PCB inspection environments. Image dimensions span from approximately 1540 × 1285 pixels to 5871 × 4754 pixels. This variability in image size and quality supports the development and evaluation of defect detection models capable of generalizing to diverse imaging conditions and PCB designs.

**Fig. 2:**
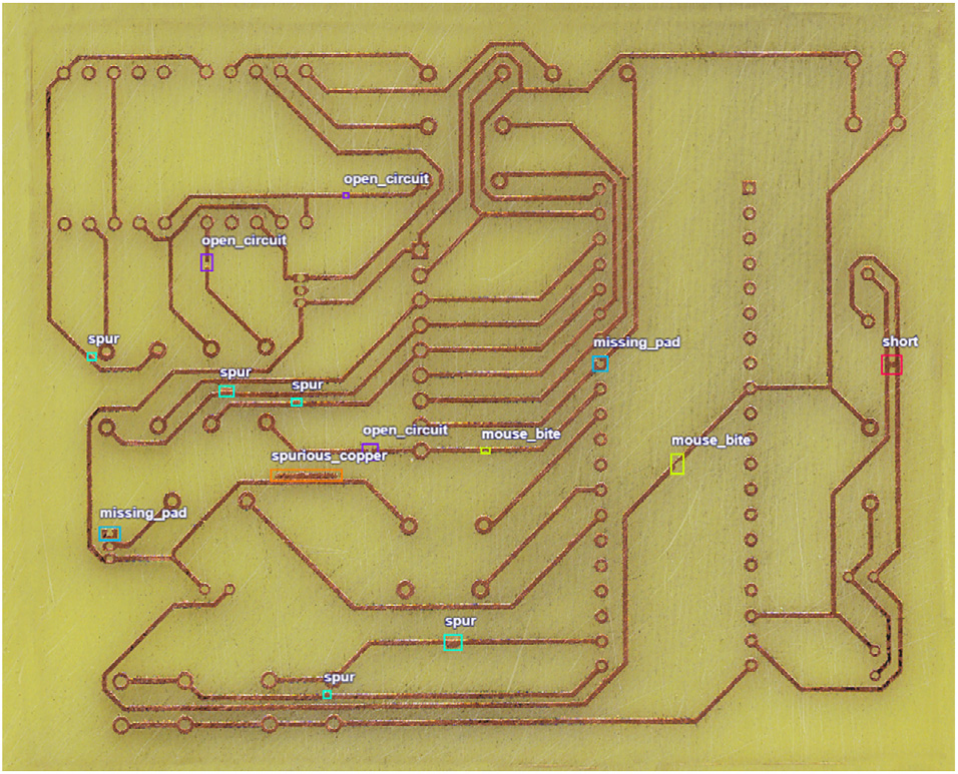
Sample diagram image containing annotations for PCB defect detection.

**Fig. 3:**
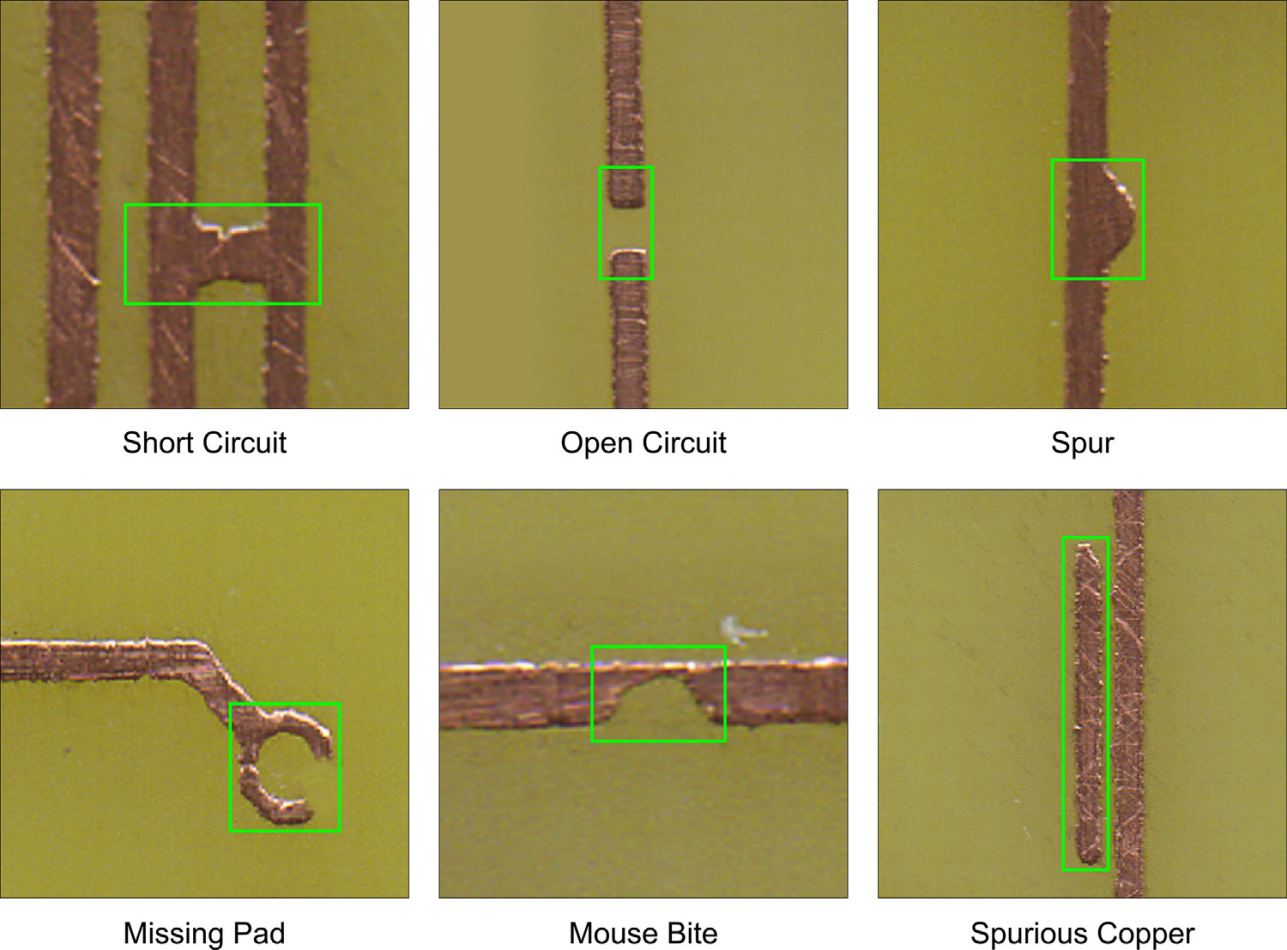
Representative Sample from each of 6 defect classes in the dataset.

To ensure comprehensive coverage of defect types, six common PCB fault classes were intentionally introduced during the design phase. These defects were then physically realized through the chemical etching process. The annotation process was conducted manually using specialized labeling tools, with each defect instance precisely localized using axis-aligned bounding boxes, as illustrated in Fig. 2. The six PCB defect classes are depicted in Fig. 3, along with example image patches showing their characteristic appearances (highlighted by green boxes). This selection of defect types draws on industrially observed faults as documented in the review by Sankar et al. [2], which identifies common defects such as track shorts, open circuits, pad-related faults including mouse bites, spurious copper, and spurs, as well as missing pads. These overlapping categories support the relevance of our dataset's chemically etched defect classes to realistic manufacturing conditions.

• **Short Circuit:** An unintended electrical connection between distinct conductors caused by excess copper or solder bridging. This allows current to flow along an undesired path, leading to device malfunction, overheating, or damage. Shorts are most often introduced by manufacturing residue or process errors [7,8].
• **Open Circuit:** A discontinuity in a circuit trace or pad, interrupting current flow. Opens can occur due to incomplete etching, broken traces, or missing connections, resulting in non-functional parts of the PCB [7,9].
• **Missing Pad:** A pad is an exposed area on the PCB for soldering components. Missing pad defects occur when portions or the entirety of a pad are absent, either due to over-etching, misaligned manufacturing processes, or mechanical damage, impeding reliable component attachment [2].
• **Spur:** A spur is a thin, unintended extension or protrusion from a trace or pad, resulting from over-etching or process inconsistencies. Such protrusions can potentially bridge gaps meant to remain isolated, risking shorts or signal integrity issues [10,11].
• **Spurious Copper:** Random, unwanted pieces of copper that remain after the etching process due to incomplete removal. Spurious copper can create unintended conductive paths, leading to shorts or unpredictable circuit behavior [12].
• **Mouse Bite:** Characterized by small, irregular notches or perforations resembling bite marks, usually at the board's edges or within conductor paths. These are often caused by imperfect depaneling (removal from the production panel) or routing, weakening the mechanical integrity and potentially affecting electrical performance [13].

The PCB images were acquired by scanning the single-layer PCBs after fabrication using an HP ScanJet Pro 3600 f1 Flatbed scanner. This device employs Contact Image Sensor (CIS) technology and sflatbed scanning at an optical resolution up to 1600 dpi for the colored PCB images. The high hardware resolution enables high-fidelity capture of PCB details. Scans were performed in 24-bit color depth with 48-bit internal processing, producing images with rich color information suitable for defect analysis.

**Fig. 4:**
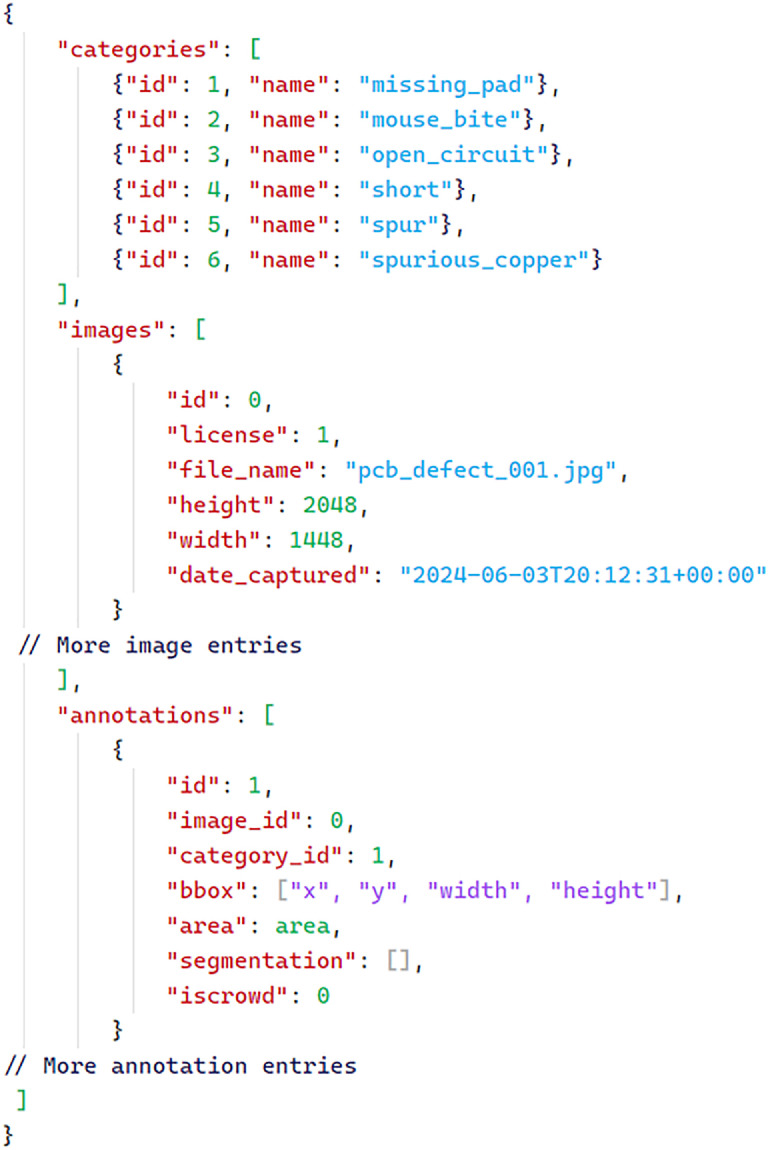
A sample JSON file consistent with the annotation format used for PCB defect classification and localization.

Table 1 summarizes the total number of annotated instances per defect class in the dataset, highlighting an average of approximately 284 annotations per class and demonstrating a balanced representation of real-world PCB faults. The dataset annotations are stored in a JSON file format adapted from the widely recognized COCO (Common Objects in Context) standard [14]. This format is designed to support efficient mapping between images, defect categories, and individual annotations, making it compatible with most modern object detection frameworks [15]. The JSON file is organized into three primary sections: categories, images, and annotations, as shown in Fig. 4.

• **categories:** This section defines the six defect classes present in the dataset. Each class is assigned a unique id and a descriptive name, such as “missing_pad”, “mouse_bite”, “open_circuit”, “short”, “spur”, and “spurious_copper”. This structure enables multi-class detection and facilitates the extension of the dataset with additional classes if needed (Table 2).

**Table 2:** Distribution of instances across different defect classes within the PCB dataset.

Total Annotations per Class	
Mouse-Bite	356
Spurious-Copper	246
Spur	296
Open-Circuit	276
Short-Circuit	254
Missing-Pad	276
• **images:** Each entry in this section corresponds to a single PCB image. Metadata for each image includes a unique id, the file_name, the image’s height and width, and the date_captured.
• **annotations:** This section contains detailed information about each defect instance. Each annotation includes a unique id, the *“image_id”* to which it belongs, the *“category_id”* indicating the defect class, and a *“bbox”* array specifying the bounding box coordinates in the format [x, y, width, height]. Additional fields such as *“area”* (the size of the bounding box), *“segmentation”* (left empty as polygonal outlines are not used), and *“iscrowd”* (set to 0, indicating individual defects) are also included.
  ○ ***“id”***: A unique identifier for each annotation instance, ensuring that every defect annotation can be individually referenced within the dataset.
  ○ ***“image_id”***: Links the annotation to a specific image by referencing the unique identifier of the image in which the defect appears; this connects each defect instance to its corresponding PCB image.
  ○ ***“category_id”***: Indicates the type of defect by referencing the unique identifier of the defect class as defined in the categories section (e.g., missing pad, open circuit).
  ○ ***“bbox”***: Specifies the location and size of the defect within the image using an array of four values: [x, y, width, height], where (x, y) are the coordinates of the top-left corner of the bounding box, and width and height are its dimensions in pixels.
  ○ ***“area”***: Represents the total area covered by the bounding box, typically calculated as width × height, and is used for quantitative analysis of defect size.
  ○ ***“segmentation”***: Contains a list of coordinates outlining the polygonal shape of the defect for more precise localization; in your dataset, this field is left empty as only bounding boxes are used for annotation.
  ○ ***“iscrowd”***: A binary flag (0 or 1) indicating whether the annotation represents a single defect instance (0) or a group of overlapping defects treated as a crowd (1); in the PCB Defect dataset, this is set to 0, as each defect is annotated individually.

**Table 1:** Scanner Settings and Image acquisition parameters for PCB Dataset.

Total Annotations per Class	
Resolution (DPI)	1600
Color Mode	24-bit color (True Color)
Exposure/Brightness	Default automatic scanner settings
Scanning area	5 × 8 inches
File Format	JPEG
Image Correction	No auto enhancements

### 3.1 Dataset directory structure

**Fig. 5:**
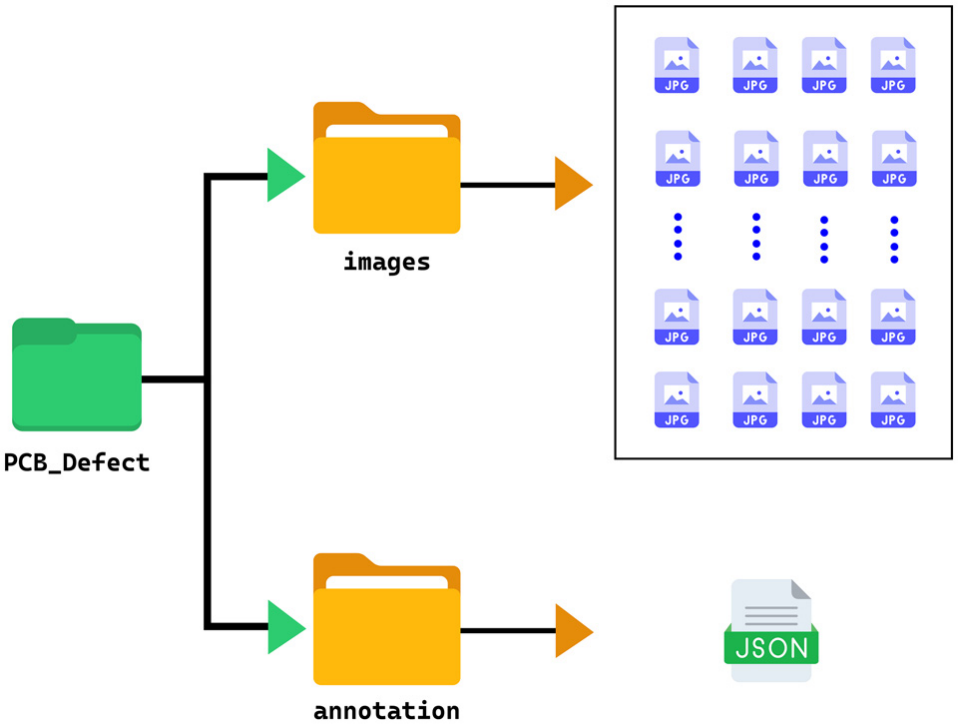
Directory structure of PCB defect detection dataset.

The PCB Defect Detection dataset is structured to provide straightforward and efficient access to both image and annotation data required for deep learning-based computer vision research. The directory structure, including the placement of the ``images'' folder and the ``pcb_annotations.json'' file, is illustrated in Fig. 5.

• *“images”:* All the PCB images are contained directly within the main directory. Each image is named using a consistent convention (e.g., “pcb_defect_001.jpg”) to facilitate traceability with corresponding annotation entries. The images vary in resolution, ranging from 800 × 600 pixels to 6000 × 4000 pixels, and have undergone preprocessing to standardize their dimensions where necessary.
• *“annotation”:* A single JSON file, named “pcb_annotations.json”, is also present in the main directory. This file follows the COCO (Common Objects in Context) annotation standard and contains comprehensive metadata for all defect annotations within the dataset.

There are no nested subdirectories; all image files and the annotation JSON are at the top level of the dataset folder.

## 4 Experimental Design, Materials and Methods

### 4.1 Designing procedure

The manufacturing of the Printed Circuit Boards (PCBs) for this dataset follows a lab-based chemical etching process where real, physically manifested defects were intentionally introduced. PCB circuit designs were first created using ECAD software tools, with all layout parameters (design rule constraints, net classes, and pre-defined specifications) detailed in Table 3 (see appended table below). These settings ensured that component footprints and electrical connections were precise, and manufacturing standards for tracks, vias, clearances, and holes were strictly observed.

**Table 3:** ECAD Design rules and parameter settings for PCB fabrication.

Design Rule Constraints	
Minimum Clearance	20 mils
Minimum track width	20 mils
Minimum connection width	0 mils
Minimum annular width	20 mils
Minimum via diameter	70 mils
Copper to hole clearance	20 mils
Copper to edge clearance	20 mils
Minimum through hole	12 mils
Hole to hole clearance	10 mils
**Net Classes**	
Clearance	20 mils
Track Width	20 mils
Via Size	70 mils
Via Hole	30 mils
**Pre-Defined Specifications**	
Track Width	20,25,30,35,40,50, 60, 80 mils
Hole Shape	Circular

Designs were exported to SVG format to preserve vector fidelity and true-to-design spatial dimensions. This vector export enabled high-resolution defect introduction: using Inkscape, defects were added as precise modifications to copper features. Faults were introduced manually on the SVG vectors by selectively editing copper tracks and pads. For example, in continuous tracks or pads, small sections were erased to create discontinuities representing open circuit faults. In regions with parallel tracks, extra pads or copper traces were added to simulate short circuit faults. Defects such as mouse bites or copper errors were created by adding or removing copper features using eraser and drawing tools. The selection of the specific parts of the track or pads where faults were introduced was done completely at random. Maintaining this digital vector format ensured that all manual edits, such as removal of pad copper (missing pad), introduction of notches (mouse bites), deliberate breaks (open circuit), bridges (short circuit), small extensions (spurs), or added copper shapes (spurious copper) were transferred perfectly to the etching process without loss of detail or introduction of artifacts.

Defect placement in the dataset was performed through random distribution across traces, pads, and copper regions. Each design was carefully reviewed to prevent overlap or clustering that could obscure individual defects. Defect sizes were defined as detailed in Table 3.

The modified layouts with embedded, physically realizable defects were then printed on A4-sized photo paper using a 600 dpi laser printer for subsequent transfer onto FR4 copper-clad boards. The manufacturing of the Printed Circuit Boards (PCBs) for this dataset follows a lab-based chemical etching process where real, physically manifested defects were intentionally introduced. PCB circuit designs were first created using ECAD software tools, with all layout parameters (design rule constraints, net classes, and pre-defined specifications) detailed in Table 3. These settings ensured that component footprints and electrical connections were precise, and manufacturing standards for tracks, vias, clearances, and holes were strictly observed.

### 4.2 Manufacturing procedure

**Fig. 6:**
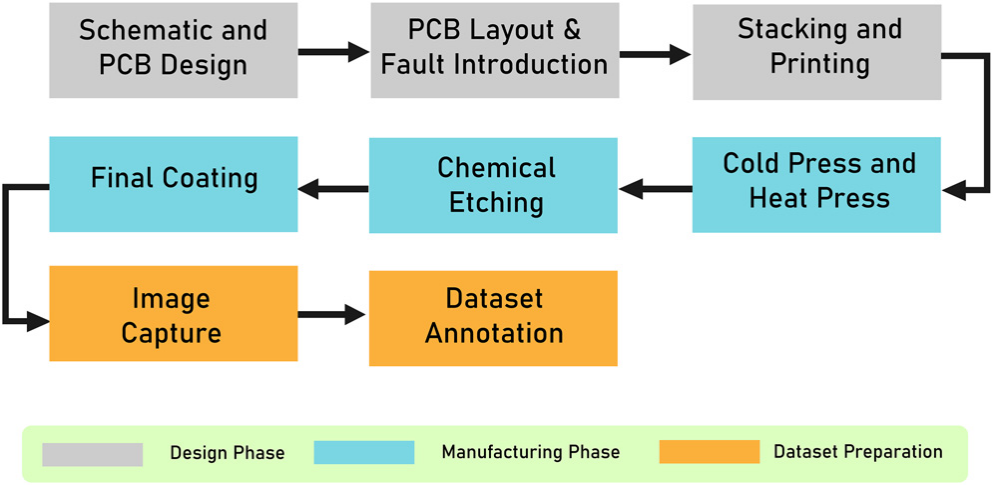
Design, Manufacturing and Processing pipeline for PCB Defect Detection Dataset.

Following the Design Procedure, the boards, fabricated using FR4 as the substrate material, were thoroughly cleaned using alcohol pads to ensure no contaminants disrupted the ink transfer. For transferring the design onto the copper-clad PCB sheets, a combination of cold and heat press methods was used. FR4, a flame-retardant woven glass-reinforced epoxy laminate, is the industry-standard material for most rigid PCBs due to its excellent mechanical strength, electrical insulation properties, dimensional stability, and cost-effectiveness [16]. Below is an outline of the primary manufacturing steps for the PCBs:

• To prepare the copper-clad FR4 sheets, uneven copper areas were carefully abraded to remove excess irregularities and ensure a smooth base for design transfer. Next, the boards were thoroughly cleaned with alcohol pads to eliminate contaminants that could disrupt ink transfer.
• During the cold press phase, the PCB design printed on A4-sized printed photo paper was imposed on the FR4 copper surface, and the copper surface was sprayed using a mixture of 60 % isopropyl alcohol and 40 % acetone. After allowing the boards to air dry, the heat press phase used a heated laminator applying approximately 25 passes, ensuring secure and smudge-free ink transfer.
• Once the design was transferred, the boards were submerged in a ferric chloride etchant solution which rapidly dissolved unmasked copper layers, leaving behind the circuit traces and pre-designed defect features. The etching process was carefully controlled by monitoring etchant concentration, temperature, and solution agitation to guarantee accuracy.
• After etching, boards were thoroughly cleaned to remove residual ink and etchant chemicals. A clear epoxy resin was applied as a protective coating, serving to improve thermal dissipation and protect against moisture and oxidation.
• Post-etching, each PCB underwent strict quality control checks to verify the proper manifestation of defects, ensuring the dataset's images accurately reflect real-world PCB manufacturing imperfections while maintaining traceability and reproducibility. The overall process workflow is summarized in Fig. 6.

### 4.3 Annotation process and quality control

The dataset includes manual annotations done primarily by a dedicated annotator using the Roboflow tool. Although no formal annotation guidelines document was prepared, the annotator followed established best practices to carefully trace bounding boxes around visible defect boundaries while minimizing inclusion of non-defective regions. Defect classes were consistently assigned according to the six defined categories.

To improve annotation reliability, the annotations underwent two rounds of sequential validation by two other experts who reviewed the labeled images for consistency, accuracy, and compliance with the defect class definitions. Any ambiguities or errors found during validations were discussed and resolved collaboratively, ensuring high annotation quality throughout the dataset.

### 4.4 Dataset preparation

**Fig. 7:**
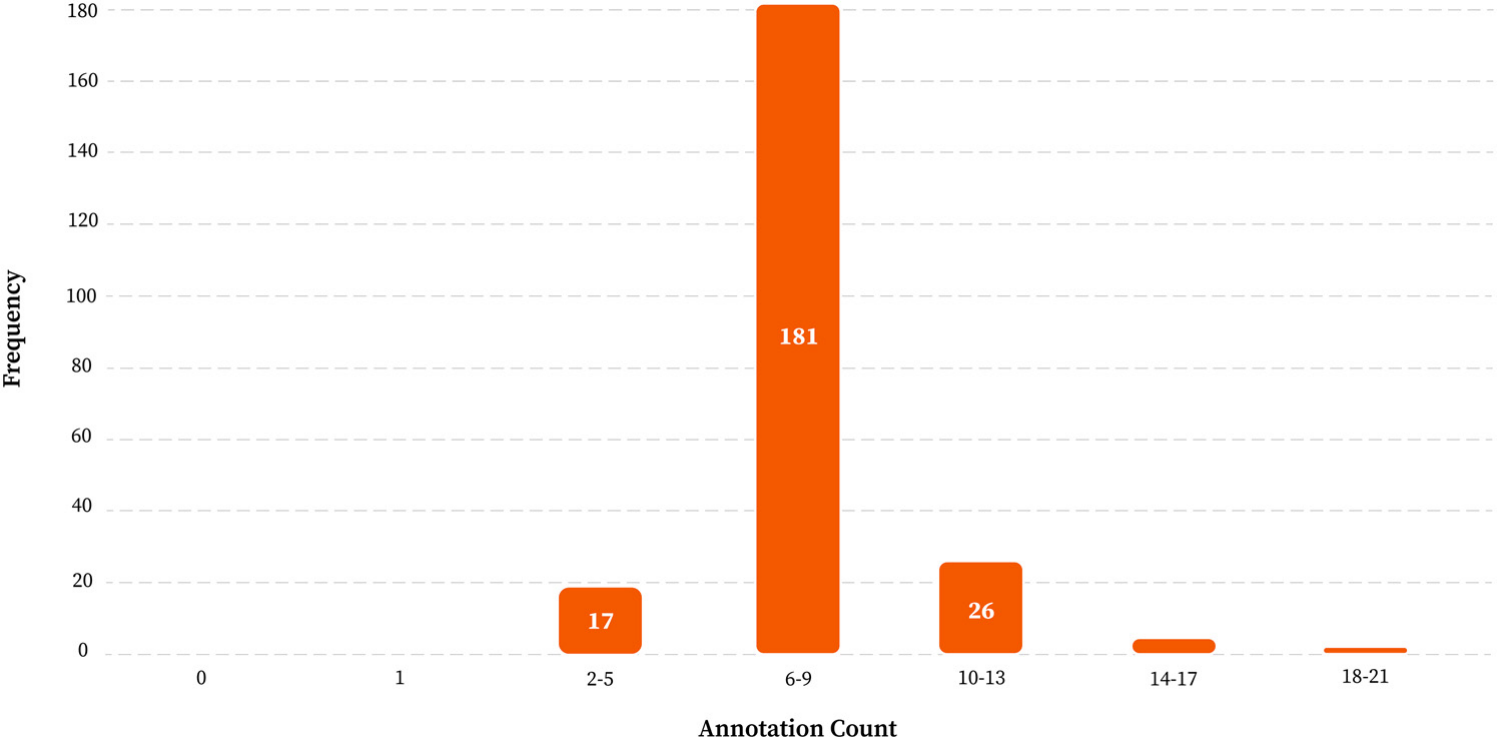
Histogram of Object Count by Image of PCB Defect Detection Dataset.

The capture of PCB images for this dataset was accomplished by scanning each single-layer PCB using the HP ScanJet Pro 3600 f1 Flatbed scanner after the boards were fabricated. Each of the 230 PCB images was annotated manually by the authors using bounding boxes to localize six defined defect types. Annotations were stored in COCO-style JSON format to ensure compatibility with modern object detection frameworks. The labels—missing pad, mouse bite, open circuit, short, spur, and spurious copper—were structurally mapped using unique category IDs. There are 1704 defect annotations which are spread on these images and the average number of defect instances per image is 7.4. This level of annotation density is representative of actual industrial practice, in which it is quite common to have several faults occurring concurrently on a single board. It has 6 classes of defects as the dataset is organized: Mouse-Bite (356 instances), Spur (296), Open-Circuit (276), Missing-Pad (276), Short-Circuit (254), and Spurious-Copper (246). The histogram shown in Fig. 7 illustrates the distribution of object counts across images, confirming that most images contain a moderate and realistic number of defect annotations.

Roboflow is an open-source platform that facilitates efficient image annotation and dataset preparation for machine learning tasks. Its use streamlined the annotation workflow and ensured compatibility with standard object detection frameworks [17]. The annotation process utilized the Roboflow annotation tool for bounding box labeling and management.

## Limitations

During the etching process, the edges of copper traces are neither completely smooth nor perfectly vertical. Rough edges, known as edge definition issues, occur due to mask resolution limitations, non-uniform acid circulation, and gas bubbling, potentially affecting defect boundaries and model accuracy.

• The dataset does not include the application of the green solder mask layer, which in real-world PCBs serves to protect copper traces and reduce solder bridging. The absence of this layer means the dataset may not fully reflect the final appearance and masking-related defects in industrial PCBs.
• This dataset represents only single-layer PCBs, whereas multi-layer PCBs are common in complex electronics. The lack of multi-layer complexity limits the dataset’s applicability for defect detection in multi-layer or more intricate PCB assemblies.
• The controlled laboratory etching environment may not capture the full range of variability found in large-scale industrial manufacturing, such as machine wear, environmental factors, or diverse substrate materials beyond FR4, possibly limiting generalization to all production settings. While these produce physical defects with realistic morphology, they may not capture the full variability or stochastic nature of defects arising in industrial mass production.
• Imaging was performed using a flatbed scanner rather than industrial camera systems typical in manufacturing lines. Although the scanner provides high-resolution, rich color images, the image acquisition conditions differ from those in automated optical inspection (AOI) environments.
• Lastly, the dataset size is moderate, containing 230 PCB images. This size provides a valuable benchmark and initial resource; however, larger datasets may be necessary to fully train state-of-the-art deep learning models aimed at production-scale deployment.

## Ethics Statement

This work does not involve human subjects, animal experiments, or any data collected from social media platforms. Therefore, ethical approval from an institutional review board or ethics committee was not required. The authors have read and fully comply with the ethical requirements for publication in Data in Brief, affirming that this study meets all applicable ethical standards.

## CRediT Author Statement

**Ahmed Jawad Rashid:** Conceptualization, Methodology, Software, Validation, Data Curation, Writing-Original Draft; **Mohammad Aman Ullah:** Conceptualization, Methodology, Software, Validation, Data Curation, Writing- Review & Editing; **Adiba Isfara:** Conceptualization, Methodology, Software, Validation, Data Curation; **Nadim Ahmed:** Conceptualization, Writing- Review & Editing, Supervision, Project administration; **Mamun Mian:** Conceptualization, Methodology, Resources; **Md. Mashur Shalehin:** Conceptualization, Software, Validation.

## Data Availability

Mendeley DataPCB-Defect: An Annotated Dataset for Surface Defect Detection in Printed Circuit Boards (Original data).
